# PREVALENCE OF CEREBRAL VENOUS DRAINAGE STENOSES ON RETROSPECTIVELY ANALYZED CTA FOR STROKE TRIAGE

**DOI:** 10.1093/geroni/igad104.0779

**Published:** 2023-12-21

**Authors:** Ferdinand Hui

**Affiliations:** Kuakini Medical Center, Honolulu, Hawaii, United States

## Abstract

Cerebral venous congestion (CVC) has been identified as a hypothetic cause of cognitive impairment. Prior work has estimated that 15-30% of the population may have jugular venous narrowing, a potential cause of CVC, based on reviews of CT angiography (CTA) performed at a large academic medical center. However, few comprehensive studies exist. Therefore, for the current work, we reviewed fifty CTAs that were obtained for ischemic stroke triage and thrombectomy planning at a comprehensive stroke center in Hawaii (Queens Hospital, Honolulu HI). These brain CTAs were analyzed for arterial and venous variants and other abnormalities that might place the patient at increased risk for arterial injury and/or venous congestion, both of which may result in cognitive decline. The CTAs were analyzed retrospectively for every known level of venous and arterial stenosis, and CVC-associated pre-morbid symptoms were analyzed. Preliminary data support the hypothesis that venous anatomical anomalies may be a risk factor for cognitive impairment. CVC may be an under-diagnosed contributor to cognitive impairment, particularly in older patients.

